# Proximal C-Terminus Serves as a Signaling Hub for TRPA1 Channel Regulation via Its Interacting Molecules and Supramolecular Complexes

**DOI:** 10.3389/fphys.2020.00189

**Published:** 2020-03-12

**Authors:** Lucie Zimova, Kristyna Barvikova, Lucie Macikova, Lenka Vyklicka, Viktor Sinica, Ivan Barvik, Viktorie Vlachova

**Affiliations:** ^1^Department of Cellular Neurophysiology, Institute of Physiology, The Czech Academy of Sciences, Prague, Czechia; ^2^Department of Physiology, Faculty of Science, Charles University, Prague, Czechia; ^3^Department of Physical and Macromolecular Chemistry, Faculty of Science, Charles University, Prague, Czechia; ^4^Division of Biomolecular Physics, Faculty of Mathematics and Physics, Institute of Physics, Charles University, Prague, Czechia

**Keywords:** TRPA1, TRP channel, calmodulin, A-kinase anchoring protein, transient receptor potential

## Abstract

Our understanding of the general principles of the polymodal regulation of transient receptor potential (TRP) ion channels has grown impressively in recent years as a result of intense efforts in protein structure determination by cryo-electron microscopy. In particular, the high-resolution structures of various TRP channels captured in different conformations, a number of them determined in a membrane mimetic environment, have yielded valuable insights into their architecture, gating properties and the sites of their interactions with annular and regulatory lipids. The correct repertoire of these channels is, however, organized by supramolecular complexes that involve the localization of signaling proteins to sites of action, ensuring the specificity and speed of signal transduction events. As such, TRP ankyrin 1 (TRPA1), a major player involved in various pain conditions, localizes into cholesterol-rich sensory membrane microdomains, physically interacts with calmodulin, associates with the scaffolding A-kinase anchoring protein (AKAP) and forms functional complexes with the related TRPV1 channel. This perspective will contextualize the recent biochemical and functional studies with emerging structural data with the aim of enabling a more thorough interpretation of the results, which may ultimately help to understand the roles of TRPA1 under various physiological and pathophysiological pain conditions. We demonstrate that an alteration to the putative lipid-binding site containing a residue polymorphism associated with human asthma affects the cold sensitivity of TRPA1. Moreover, we present evidence that TRPA1 can interact with AKAP to prime the channel for opening. The structural bases underlying these interactions remain unclear and are definitely worth the attention of future studies.

## Introduction

The Transient Receptor Potential (TRP) Ankyrin subtype 1 (TRPA1), originally called ANKTM1 (Story et al., 2003), is a cation channel expressed in a subset of dorsal root, trigeminal and visceral primary sensory neurons (Bautista et al., 2006; Kwan et al., 2006), but also in non-neuronal cells such as keratinocytes (Kwan et al., 2006), fibroblasts (Jaquemar et al., 1999), odontoblasts (El Karim et al., 2011), astroglia (Shigetomi et al., 2011), Schwann cells (De Logu et al., 2017), endothelial cells, and arterial vessels (Kwan et al., 2009). There, TRPA1 acts as a polymodal sensor of cell threats, being activated by a wide range of physical and chemical stimuli of extracellular or intracellular origin (for comprehensive reviews see (Zygmunt and Hogestatt, 2014; Chen and Hackos, 2015; Viana, 2016; Gouin et al., 2017; Koivisto et al., 2018; Wang et al., 2019) and references therein). Accumulating evidence links the physiological functions of TRPA1 to inflammation, temperature perception, mechanosensation, insulin secretion, itching, respiratory functions, regulation of the cardiovascular system, but also the homeostatic balance between the immune and nociceptive systems, as recently nicely reviewed by Talavera et al. (2019). Under certain pathological conditions such as tissue injury or inflammation, TRPA1 may undergo a wide range of posttranslational modifications that lead to various levels of functional modulation. Ca^2+^ influx through TRPA1 can release mediators such as calcitonin gene-related peptide, substance P, neurokinin A and bradykinin, which modulate the channel via G-protein-coupled receptor signaling cascades (Andrade et al., 2012; Petho and Reeh, 2012; Voolstra and Huber, 2014; Kadkova et al., 2017) and/or promote the recruitment of the channels to the cell surface (Schmidt et al., 2009; Takahashi and Ohta, 2017).

A large number (>150) of single-point mutations and chimeras of human TRPA1 have been functionally characterized in the literature (Meents et al., 2019), yet the published data do not enable us to fully understand the molecular details of the channel function, mostly because (1) the available structures of TRPA1 capture the channel in an intermediate or inactivated conformation (Paulsen et al., 2015; Suo et al., 2020) which does not allow to distinguish the functional states, (2) the structures lack information on a considerable (∼50%) part of the protein, (3) the impact of the interactions of TRPA1 with various important endogenous proteins and cellular signaling mechanisms has not yet been fully characterized (Zygmunt and Hogestatt, 2014; Gouin et al., 2017; Talavera et al., 2019), and (4) the extent to which TRPA1 can be regulated by its membrane environment is still only gradually being uncovered (Hirono et al., 2004; Akopian et al., 2007; Dai et al., 2007; Karashima et al., 2008; Witschas et al., 2015; Macikova et al., 2019; Startek et al., 2019). Obviously, a better understanding of all these issues is key for a precise description of the mechanisms of TRPA1 activation, and, perhaps more importantly, for rational screening of its novel modulators as potential therapeutic agents.

The thermosensitive properties of TRPA1 are even less understood. In mammals, TRPA1 is thought to function as a cold detector (Story et al., 2003; Viswanath et al., 2003; Bandell et al., 2004; Kwan et al., 2006; Karashima et al., 2009; Kremeyer et al., 2010), but it has been also found to play a crucial role in the detection of noxious heat (Hoffmann et al., 2013; Yarmolinsky et al., 2016; Vandewauw et al., 2018). *In vitro*, a direct cold activation of TRPA1 was demonstrated by several laboratories for mouse, rat and human orthologs (Story et al., 2003; Viswanath et al., 2003; Sawada et al., 2007; Karashima et al., 2009; del Camino et al., 2010; Moparthi et al., 2016). On the other hand, some other groups did not observe any cold activation (Jordt et al., 2004; Zurborg et al., 2007; Knowlton et al., 2010; Cordero-Morales et al., 2011; Chen et al., 2013). This is clearly not the whole story, and further intensive investigation is required to determine the specific role of mammalian TRPA1 as a temperature sensor (Sinica et al., 2019).

This article provides new evidence that the cold sensitivity of TRPA1 can be modulated by membrane phosphoinositides; specifically, by phosphatidylinositol-4,5-bisphosphate, PIP_2_. Moreover, we demonstrate that AKAP, the scaffolding A-kinase anchoring protein that is necessary for the effective phosphorylation of TRPA1 by protein kinases A and C, potentiates the channel at negative membrane potentials, suggesting the existence of basal phosphorylation or a direct effect of AKAP on TRPA1. Although these primary results provide potentially important information indicating that the membrane proximal part of the C-terminus of TRPA1 may form a hot spot contributing to a highly effective regulation of TRPA1, additional structural/functional considerations are necessary to characterize the channel in its full physiological context.

## Materials and Methods

### Cell Culture, Constructs, and Transfection

Human embryonic kidney 293T (HEK293T; ATCC, Manassas, VA, United States) cells were cultured and transfected with 400 ng of cDNA plasmid encoding wild-type or mutant human TRPA1 (pCMV6-XL4 vector, OriGene Technologies, Rockville, MD, United States) and 200 ng of GFP plasmid (TaKaRa, Shiga, Japan), and, for particular experiments, 300 ng of a plasmid of wild-type TRPA1 with 300 ng of plasmid Dr-VSP (in IRES2-EGFP vector, a gift from Yasushi Okamura, Addgene plasmid #80333) or 400 ng of the plasmid of wild-type TRPA1 with 200 ng of plasmid AKAP79 (pCMV6-XL4 vector, OriGene Technologies, Rockville, MD, United States), using the magnet-assisted transfection technique (IBA GmbH, Gottingen, Germany) as described previously (Zimova et al., 2018). The mutant H1018R was generated by PCR using a QuikChange II XL Site-Directed Mutagenesis Kit (Agilent Technologies, Santa Clara, CA, United States) and confirmed by DNA sequencing (Eurofins Genomics, Ebersberg, Germany).

### Electrophysiology and Cold Stimulation

**FIGURE 1:**
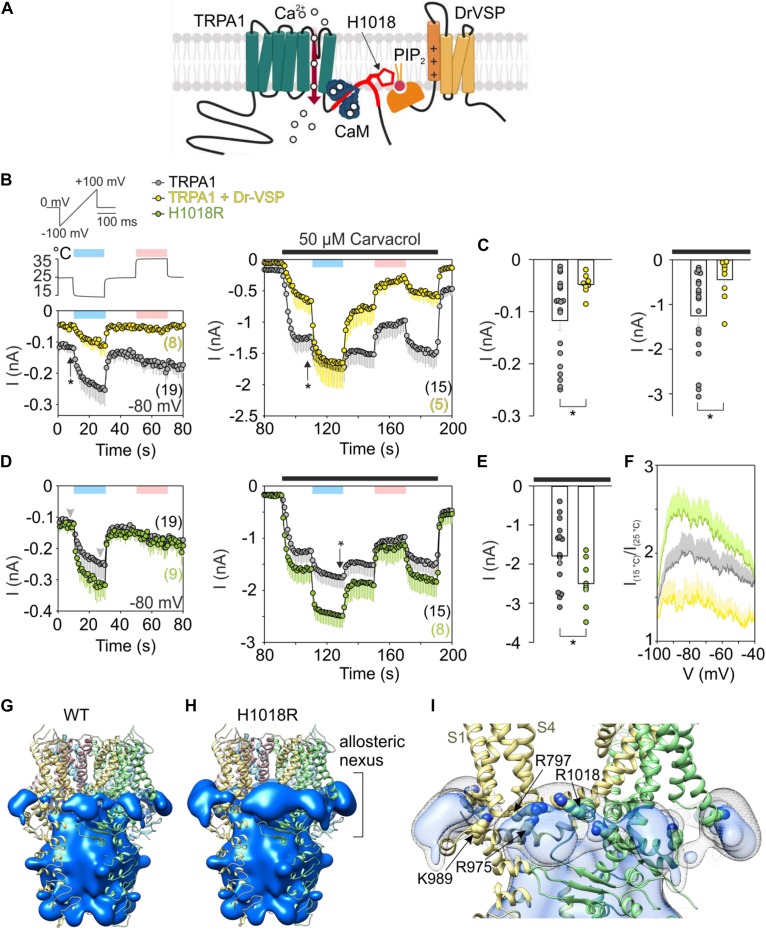
Acute PIP_2_ depletion has an opposite effect on cold-dependent gating of TRPA1 to missense histidine-to-arginine mutation at position 1018. **(A)** Schematic diagram shows binding of Ca^2+^-CaM (calmodulin) to C terminus of TRPA1 (Macikova et al., 2019). The binding domain for CaM partly overlaps with the proposed binding pocket for PIP_2_ (in red) which includes H1018. Voltage-sensitive phosphatase (Dr-VSP) hydrolyses PIP_2_ upon depolarization greater than +50 mV. **(B)** Time course of average whole-cell currents of human TRPA1 (TRPA1, gray circles with bars indicating mean values with – SEM) and TRPA1 co-expressed with Dr-VSP (yellow circles with bars indicating mean values with – SEM), measured at –80 mV. First, the cells were exposed to a 3 s depolarizing pulse to +80 mV to activate Dr-VSP. Then, the membrane potential was linearly ramped up each second from –100 mV to +100 mV (1 V.s^– 1^). The temperature was lowered first from room temperature 25°C to 15°C (blue bar) and then raised to 35°C (pink bar); the temperature trace is shown above the first record (left). Subsequently, 50 μM carvacrol (black bar) was applied together with temperature changes (right), n is indicated in brackets. **(C)** Comparison of current amplitudes at –80 mV at times marked in **(B)** with vertical arrows. Color coding as in **(B)**. Left: The basal current through TRPA1 is significantly smaller (*P* = 0.028) when it is co-expressed with Dr-VSP that causes acute PIP_2_ depletion upon voltage stimulation. Right: Responses to carvacrol are also significantly smaller (*P* = 0.032). Data are shown as single points and as mean values – SEM. **(D)** Time course of average whole-cell currents of mutant H1018R of TRPA1 (green circles with bars indicating mean value – SEM) measured at –80 mV compared with wild-type hTRPA1 as in **(B)**. **(E)** The current responses of H1018R to simultaneous exposure to 50 μM carvacrol and cold (15°C) are significantly higher (*P* = 0.043) than the currents from wild-type hTRPA1. Data are shown as single points and as mean values – SEM at times marked in **(D)** with an arrow. Color coding as in **(B,D)**. **(F)** Voltage dependence of cold activation averaged at times indicated by gray arrowheads in **(D)**. The extent of cold potentiation of TRPA1 (gray line, mean + SEM), hTRPA1 co-expressed with Dr-VSP (yellow line, mean + SEM), and H1018R (green line, as mean + SEM) at negative membrane potentials. **(G)** Areas of positive electrostatic potential (blue surface) surrounding the ligand-free structure of TRPA1 [Protein Data Bank (PDB) ID: 6PQQ] and **(H)** its mutant H1018R. **(I)** A detailed view of the region around the allosteric nexus of TRPA1 shows substantially more positive values for H1018R (side chain shown) than for TRPA1 (depicted as light gray mesh encircling light blue surface). The allosteric nexus formed by the cytoplasmic region situated below the transmembrane core has been recently proposed to be an important determinant for phospholipid binding as well as for TRPA1 gating (Zhao et al., 2019; Suo et al., 2020).

**FIGURE 2:**
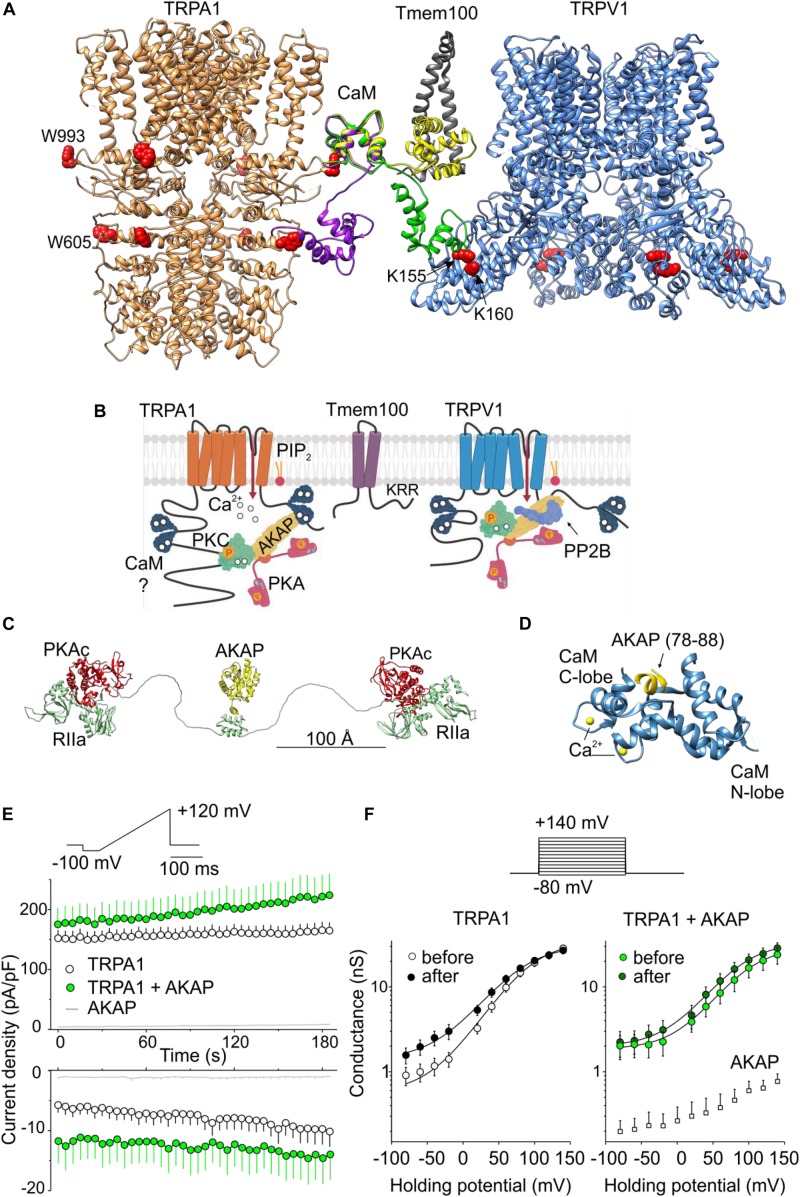
Regulation of TRPA1 by phosphorylation and by predicted interactions with modulatory and scaffold proteins. **(A)** N- and C-lobes of Ca^2+^/CaM (three conformers are shown in violet, green, and yellow) are capable of binding to TRPA1 but also serving as a linker binding either TRPA1 to the Tmem100 (gray ribbon) membrane protein or to the cytoplasmic binding site in the N-terminus of TRPV1. Tryptophans putatively involved in Ca^2+^/CaM binding are shown as red side chains. **(B)** Permeating Ca^2+^ binds to calmodulin (forming a complex Ca^2+^/CaM), phosphatidylinositol-4,5-bisphosphate (PIP_2_) competes with Ca^2+^/CaM for binding to TRPA1. A kinase anchoring protein AKAP79/150 (AKAP) binds to TRPA1 and functions as a signaling scaffold for protein kinase A (PKA) and C (PKC). The activation of PKA or PKC sensitizes TRPA1 by phosphorylation (P in orange circles). The interaction of TRPA1 with TRPV1 is regulated by the transmembrane adaptor protein Tmem100 via the amino acid KRR triplet on the C-terminus of Tmem100. AKAP also binds to TRPV1 and associates with protein phosphatase 2B (PP2B, calcineurin) to effectively dephosphorylate the channel. **(C)** Extended linear configuration (the end-to-end length ∼385 Å) of pseudo-atomic model of pentameric protein assembly of AKAP (AKAP18γ, residues 88–317) connected to regulatory subunits (RIIα) with associated catalytic subunits (PKAc) of PKA [PDB ID: 3j4R; (Smith et al., 2013)]. **(D)** Published crystal structure of CaM (steel blue ribbon) in complex with AKAP79 peptide (yellow). Two EF hands of the C-lobe coordinate Ca^2+^ (yellow atoms). One of the two copies of the published structure is shown: PDB ID: 5NIN, chains **A** and **C**. **(E)** Average whole-cell current densities induced by depolarizing voltage measured from HEK293T cells expressing TRPA1 (white circles indicating mean ± SEM; *n* = 17), or TRPA1 together with AKAP79 (green circles indicating mean ± SEM; *n* = 13). Voltage ramp protocol (shown in upper trace) was applied repeatedly each 5 s for 3 min. Amplitudes were measured at –100 mV and +100 mV and plotted as a function of time. Mean current densities measured from HEK293T cells transfected only with AKAP79 and control plasmid are shown as light gray lines with ± SEM (*n* = 4); absent error bars are smaller than the line thickness. **(F)** Average conductances measured at the end of pulses from currents induced by voltage-step protocol (shown above): 100-ms steps from –80 mV to +140 mV (increment +20 mV), holding potential –70 mV. Currents were measured from TRPA1 or TRPA1 together with AKAP79 before (white and light green circles) and after (black and dark green circles) the train of ramp pulses as shown in **(E)**. The data represent the mean ± SEM (*n* = 12 and 9, respectively). The solid lines represent the best fit to a Boltzmann function as described in (Zimova et al., 2018). Average conductances obtained from three HEK293T cells transfected with AKAP79 together with control plasmid are shown as white squares +SEM. Using depolarizing voltage ramps from –100 mV to +100 mV under whole-cell patch clamp conditions, we measured TRPA1-mediated responses from HEK293T cells transfected with wild-type human TRPA1 or wild-type human TRPA1 together with the voltage-dependent phosphatase cloned from *Danio rerio* (Dr-VSP) to selectively deplete PIP_2_ (Hossain et al., 2008; Okamura et al., 2009; Zimova et al., 2018; Figure 1A). We applied 20 s steps from 25°C to 15°C and to 35°C first in control extracellular solution and then in 50 μM carvacrol, the non-electrophilic agonist of TRPA1. At positive membrane potentials the current responses were not significantly different, indicating comparable expression levels. The comparison at −80 mV indicates that an acute level of membrane PIP_2_ regulates both the rate and extent of the response to individual stimuli (cold temperature or agonist) but does not affect the synergy between the stimuli (Figures 1B,C). Using the same voltage protocol, we measured currents from cells expressing the H1018R mutant (Figure 1D). Notably, the H1018R mutant exhibited significantly increased current responses at −80 mV upon the simultaneous application of agonist and cooling (Figure 1E). The voltage dependence of cold activation (Figure 1F) illustrates the opposite effects of the H1018R mutation and the acute PIP_2_ depletion. To find where negatively charged PIP_2_ will most likely be bound, we used the structure of human TRPA1 [Protein Data Bank (PDB) ID: 6PQQ; (Suo et al., 2020)]. By means of the “Mutate residue” plugin in VMD software (Humphrey et al., 1996), H1018 was mutated to R1018. The “PME Electrostatics” module of VMD was used to compute electrostatic maps, which were visualized with UCSF Chimera (Pettersen et al., 2004). As shown in Figures 1G–I, the H1018R mutation substantially extended the positive electrostatic potential surrounding the allosteric nexus formed by the cytoplasmic region situated just below the transmembrane core, indicating an increased probability of PIP_2_ binding (see also the Supplementary Material). These results further substantiate the previously proposed role of the proximal C-terminal linker in the PIP_2_-mediated regulation of TRPA1, and also indicate that the cold sensitivity of the channel can be modulated by membrane lipids. Consistent with our results, the allosteric nexus containing this region has been recently proposed to be an important determinant for phospholipid binding as well as for TRPA1 gating (Zhao et al., 2019; Suo et al., 2020). At present, however, it is not clear to what extent the results may reflect the situation *in vivo* and how the effects observed with the H1018R mutant may relate to human asthma. We have recently demonstrated that the cold sensitivity of human TRPA1 is similar in both HEK293T cells and also in F11 cells, which are a well characterized cellular model of peripheral sensory neurons (Sinica et al., 2019). Although the TRPA1-mediated cold responses do not differ between these two cellular models, PIP_2_ signaling and H1018R expression could differ among various cell types.

All electrophysiological recordings were carried out as described previously (Zimova et al., 2018). For the experiments described in Figure 1, the extracellular bath solution contained: 140 mM NaCl, 5 mM KCl, 2 mM MgCl_2_, 5 mM EGTA, and 10 mM HEPES, 10 mM glucose, pH 7.4 was adjusted with TMA-OH. The intracellular solution contained 140 mM KCl, 5 mM EGTA, 2 mM MgCl_2_, and 10 mM HEPES, adjusted with KOH to pH 7.4. For the experiments shown in Figure 2, the extracellular bath solution contained: 140 mM NaCl, 4 mM KCl, 1 mM MgCl_2_, 10 mM HEPES, 5 mM glucose, pH 7.4 adjusted with NaOH. The intracellular solution contained 125 mM Cs-glucono-δ-lactone, 15 mM CsCl, 5 mM EGTA, 0.5 mM CaCl_2_, 2 mM MgATP, 0.3 NaGTP, and 10 mM HEPES, adjusted to pH 7.4 with CsOH. A system for the rapid cooling and heating of solutions superfusing isolated cells under patch-clamp conditions was used as described in Dittert et al. (2006). The temperature of the flowing solution was measured with a miniature thermocouple inserted into the common outlet capillary near to its orifice, which was placed less than 100 μm from the surface of the examined cell. Statistical significance was determined by Student’s *t*-test or the analysis of variance, as appropriate; differences were considered significant at *P* < 0.05 where not stated otherwise. The data are presented as means ± (or +/−) SEM.

## Results and Discussion

### Predicted Role of Phosphoinositides in the Regulation of Temperature–Dependent Gating of TRPA1

The transmembrane domain of TRPA1 is composed of the voltage sensor-like domain (VSLD) formed by a bundle of four antiparallel helices, S1–S4, and the pore domain (formed by S5, S6, and two pore helices) arranged in a domain-swap manner. The proximal C terminus contains a TRP-like domain that interacts with a pre-S1 helix. There are at least two sites with separate functions from which the activity of TRPA1 can be regulated by membrane phosphatidylinositol-4,5-bisphosphate (PIP_2_) or other phosphoinositides: the first, formed by the intracellular part of VSLD and contributed to by K989 from the TRP-like domain (Samad et al., 2011; Witschas et al., 2015; Zimova et al., 2018), and the second localized between adjacent subunits (T1003–P1034), capable of directly affecting the gating of the channel through the S4-S5 linker and R975 from the TRP-like domain (Macikova et al., 2019). Mutation at the highly conserved phenylalanine F1020 located in the center of the latter interacting region produced channels with faster activation kinetics and with significantly suppressed responses at negative membrane potentials. The effectiveness of PIP_2_ at inhibiting or promoting the activity of TRPA1 appears to substantially depend on the conformational states of the channel (Macikova et al., 2019). Importantly, two amino acids upstream of F1020, a missense mutation of a histidine residue (rs959976) was recently found to be associated with childhood asthma (Gallo et al., 2017). The histidine-to-arginine mutation (H1018R) increased the responses of recombinant channels to insoluble coal fly ash particles by 70%, suggesting an increased sensitivity to mechanical stimuli (Deering-Rice et al., 2015). We hypothesized that arginine at position 1018 may alter the affinity of PIP_2_ to TRPA1, and thereby also influence the activation of the channel by other stimuli, particularly by heat or cold temperatures. Several structures of thermosensitive TRP channels contain lipids in the regions essentially involved in agonist binding or pore gating (Cao et al., 2013; Singh et al., 2018, 2019), and it has been proposed that the association/dissociation of lipids may be one of the underlying mechanisms of temperature sensation (Cao et al., 2013). Also, recent molecular dynamics simulations performed with the structure of the TRPA1-related channel TRPV1 at different temperatures suggest that the lipid displacement from protein binding sites may contribute to temperature-evoked actuation (Melnick and Kaviany, 2018). Because human TRPA1 is considered to be a cold-sensitive channel (Moparthi et al., 2014), we tested whether the H1018R mutation may influence the cold sensitivity of human TRPA1.

### Regulation of TRPA1 by Ca^2+^-Calmodulin Complex

One of the most essential modulators of TRPA1 are calcium ions, which activate the channel at low concentrations and inactivate it at higher concentrations (Jordt et al., 2004; Nagata et al., 2005; Doerner et al., 2007; Zurborg et al., 2007; Cavanaugh et al., 2008; Wang et al., 2011). Mechanistically, Ca^2+^ ions permeating through the TRPA1 channel bind the Ca^2+^-sensing protein calmodulin (CaM), which pre-associates with the membrane proximal C-terminal region of TRPA1 (L992-N1008) from where the Ca^2+^-CaM enables the channel to distinctly respond to diverse Ca^2+^ signals (Hasan et al., 2017). It does so in a bimodal manner so that it potentiates TRPA1 at low concentrations of cytosolic Ca^2+^ and inactivates the channel at higher Ca^2+^ concentrations. The proposed Ca^2+^-CaM-binding region at TRPA1 is integrated with a putative three-stranded β-sheet formed by two anti-parallel β-strands from the N-terminus and a contacting strand that follows the C-terminal TRP-like helix. The latter, peripherally exposed β-strand binds the carboxy-lobe (C-lobe) of calmodulin and even under resting concentrations of Ca^2+^ (∼100 nM) forms a tight complex with the channel (Hasan et al., 2017). As is seen from our structural comparisons shown in Figures 1G–I and from our previous results (Macikova et al., 2019), this region overlaps with the interaction site for membrane PIP_2_ with which Ca^2+^-CaM is likely to compete.

Whereas the C-lobe of CaM acts as an effector mediating Ca^2+^-dependent gating and a tether anchoring CaM to the binding site at TRPA1, the N-lobe of CaM is only partly involved in binding and does not affect the channel gating (Hasan et al., 2017). What could the additional role of the N-lobe in TRPA1 regulation be? CaM is a well-studied ubiquitously expressed protein involved in the regulation of a large number of membrane and cytoplasmic proteins (Ishida and Vogel, 2006) and its role as a Ca^2+^-dependent modulator can be predicted with a relatively high degree of confidence (Yap et al., 2000; Mruk et al., 2014). Much less is known about its role as a Ca^2+^-dependent protein linker and a regulator of scaffold proteins (Villalobo et al., 2018). Structural comparisons shown in Figure 2A indicate that the N-lobe of CaM may either bridge different domains of TRPA1 or link the channel with some different target protein(s). This is possible due to the fact that its two independently folded Ca^2+^-binding lobes are able to interact differentially and, to some degree, separately. From the sequence analysis of human TRPA1 (Yap et al., 2000; Mruk et al., 2014), several putative CaM binding sites are predicted. Of these, the N-terminal regions K578-D606 and L488–S510, and the C-terminal region K988–K1009 have the highest score. Interestingly, the interaction of the N-lobe of CaM with the N-terminal region of TRPA1 may depend on the conformational state of the channel. Whereas the N-lobe of Ca^2+^/CaM can interact with W605 in the TRPA1 structure 3J9P obtained in the presence of allyl isothiocyanate (Paulsen et al., 2015), this tryptophan is inaccessible in the recently published structures 6PQQ, 6PQP, and 6PQO, obtained, respectively, as an apo-structure and in the presence of the reversible and irreversible electrophilic agonists BITC and JT010 (Suo et al., 2020).

### Regulation of TRPA1 by Protein-Protein Interactions

In a large subset of sensory neurons, TRPA1 physically and functionally interacts with the structurally related vanilloid receptor subtype 1 channel TRPV1 (Story et al., 2003; Akopian et al., 2007; Salas et al., 2009; Staruschenko et al., 2010; Akopian, 2011; Fischer et al., 2014; Patil et al., 2020; Figure 2B). These two channels may desensitize or sensitize each other via the elevation of intracellular calcium ions (Jordt et al., 2004; Akopian et al., 2007). In addition, a direct association of TRPA1 with TRPV1 strongly inhibits the responses of TRPA1 to electrophilic agonists, independently of Ca^2+^ (Staruschenko et al., 2010; Fischer et al., 2014). In peptidergic neurons, the interaction of these two channels is tightly regulated by the transmembrane adaptor protein Tmem100, which loosens their association and thereby releases TRPA1 from inhibition. Structurally, the regulation of this interaction depends on a KRR amino acid triplet on the C-terminus of Tmem100 (Weng et al., 2015). Importantly, the highly positively charged C-terminus of Tmem100 also contains a putative CaM binding site predicted with high confidence (L99–L115). Thus, the N-lobe of CaM can bind to the N-terminus of TRPA1 but can also bind to Tmem100 or TRPV1.

In Figure 2A, we illustrate how CaM could in principle be capable of bridging two binding sites in the TRPA1 structure or serve as a linker binding TRPA1 to the Tmem100 membrane protein or to the cytoplasmic binding site in the N-terminus of TRPV1. We used the crystal structures TRPA1 (PDB ID: 3J9P), TRPV1 (3J5P), Tmem141 (2LOR), and CaM (1MUX - a set of 30 structures determined by NMR). The C-domains of several CaM conformers from the 1MUX set were placed close to W993 in TRPA1 (red spheres), which is likely to serve as a hydrophobic anchor in the experimentally confirmed L992–N1008 binding site of CaM in the proximal C-terminal region of TRPA1 (Hasan et al., 2017). The distance between W993 and W605 (in the second putative binding site for CaM in TRPA1) roughly corresponds to the distance of the N- and C-terminal subunits in the conformer of CaM (depicted as a violet ribbon). The green conformer of CaM indicates how it could potentially bridge known binding sites in TRPA1 and TRPV1 (Lau et al., 2012). Another structure of CaM (yellow) shows the N-terminal subunit returning to the membrane, where it could be anchored by Tmem100 disturbing the TRPA1-TRPV1 complex. Interestingly, recently, the N- (amino acids 220-260) and C- (684-720) terminal domains on TRPV1 responsible for TRPA1-TRPV1 complex formation were identified (Patil et al., 2020). These domains partially overlap with the previously identified binding sites for CaM/PIP2 (189-222/682-725) (Rosenbaum et al., 2004; Ufret-Vincenty et al., 2011). This provides further support for our hypothesis that CaM could serve as linker between TRPA1 and TRPV1.

### Protein Kinase A Anchoring Protein 79/150 (AKAP) Interacts With TRPA1

It has been demonstrated that TRPA1 can be sensitized by protein kinase A (PKA), protein kinase C (PKC), cyclin-dependent kinase 5, and by early signaling events linked to Ca^2+^-dependent phosphoinositide-specific phospholipase C (PLC) enzymes that hydrolyze PIP_2_ in the inner membrane leaflet (Bandell et al., 2004; Bautista et al., 2006; Dai et al., 2007; Wang et al., 2008; Hynkova et al., 2016; Brackley et al., 2017; Meents et al., 2017; Hall et al., 2018; Sulak et al., 2018; Figure 2B). For effective phosphorylation by PKA and PKC, TRPA1 needs the presence of a scaffolding protein, AKAP (Brackley et al., 2017), that is also required for the PKA phosphorylation of TRPV1 (Zhang et al., 2008). AKAP directly interacts with TRPA1 (Zhang et al., 2008), but it also engages in multiple protein-protein interactions including Ca^2+^-CaM (Faux and Scott, 1997; Patel et al., 2017). Given the recently proposed importance of the AKAP-PKA pathway in TRPA1-mediated mechanical allodynia and cold hyperalgesia (Brackley et al., 2017; Miyano et al., 2019), it would be particularly important to test the hypothesis that AKAP may serve as a molecular hub that contributes to the specificity and efficiency of the cellular signaling network regulating TRPA1 under various physiological or pathophysiological conditions (Figures 2A–D). Although AKAP spatially constrains phosphorylation by PKA, the regulatory subunits of PKA are capable of providing an ∼16 nanometer radius of motion to the associated catalytic subunits [(Smith et al., 2013) and Figure 2C] and, therefore, the pathways regulating TRPA1 and TRPV1 may together form a supramolecular signaling complex.

Previous co-immunoprecipitation studies confirmed the interaction of AKAP with TRPA1 in HEK293 cells (Zhang et al., 2008) and in cultured trigeminal neurons (Brackley et al., 2017). We asked whether the overexpression of AKAP in HEK293T cells may influence the functional response of TRPA1. We measured currents induced by repeated depolarizing voltage ramps or steps from cells expressing TRPA1 or TRPA1 together with AKAP (Figures 2E,F). In TRPA1-expressing cells, we observed gradual current increases at negative and at positive membrane potentials (*P* < 0.001 and *P* = 0.005, respectively; *n* = 17). In cells co-expressing TRPA1 and AKAP, significant basal currents were observed at negative potentials and they did not further increase upon repeated stimulation (*P* = 0.111; *n* = 13). The expression of AKAP did not affect endogenous currents from HEK293T cells. These data can be interpreted in at least three ways: (1) AKAP may recruit TRPA1 to the plasma membrane, (2) AKAP may increase the activity of TRPA1 by increasing basal phosphorylation, and (3) the interaction with AKAP induces a conformational change that primes the channel for activation. Future, more thorough structural and functional studies that resolve the underlying mechanism may help our understanding of TRPA1 regulation and could possibly identify new targets that activate or inhibit TRPA1 for therapeutic purposes. A future direction in the search for effective treatment of asthma or mechanical and cold hyperalgesia could be to focus on a pharmacology directed toward the interacting regions of the TRPA1 channel with phospholipids and with its partner proteins, or toward the interacting proteins themselves.

## Data Availability Statement

The Supplementary Material contains the Particle Mesh Ewald electrostatic potential maps for the wild-type human TRPA1 (PDB ID: 6PQQ) and the R1018 mutant, visualized in UCSF Chimera 1.13. Other raw data supporting the conclusions of this manuscript will be made available by the authors, without undue reservation, to any qualified researcher.

## Author Contributions

VV and LZ conceptualized the study. VS, LZ, VV, KB, and IB contributed to formal analysis. VS, LM, LZ, KB, and IB investigated the study. VV, LZ, IB, and LV supervised the study. VV contributed to writing – original draft of manuscript preparation. VV, LZ, and LV contributed to writing, reviewing, and editing. All authors contributed to manuscript revision, read, and approved the submitted version.

## Conflict of Interest

The authors declare that the research was conducted in the absence of any commercial or financial relationships that could be construed as a potential conflict of interest.
